# Retrospective analysis of 300 microbial cell-free DNA sequencing results in routine blood stream infection diagnostics

**DOI:** 10.3389/fcimb.2025.1504262

**Published:** 2025-01-30

**Authors:** Claudio Neidhöfer, Niklas Klein, Aylin Yürüktümen, Tessa Hattenhauer, Rebekka Mispelbaum, Christian Bode, Tobias A. W. Holderried, Achim Hoerauf, Marijo Parčina

**Affiliations:** ^1^ Institute of Experimental Hematology and Transfusion Medicine, University Hospital Bonn, Bonn, Germany; ^2^ Institute of Medical Microbiology, Immunology and Parasitology, University Hospital Bonn, Bonn, Germany; ^3^ Department of Microbiology and Hospital Hygiene, Bundeswehr Central Hospital Koblenz, Koblenz, Germany; ^4^ Department of Internal Medicine II, University Hospital Bonn, Bonn, Germany; ^5^ Department of Hematology, Oncology, Stem Cell Transplantation, Immune and Cell Therapy, Clinical Immunology and Rheumatology, University Hospital Bonn, Bonn, Germany; ^6^ Department of Anesthesiology and Intensive Care Medicine, University Hospital Bonn, Bonn, Germany

**Keywords:** sepsis, bacteremia, blood culture, molecular diagnostic techniques, next generation sequencing, clinical metagenomics, cfDNA, microbial cfDNA

## Abstract

**Introduction:**

Bloodstream infections are a critical challenge worldwide due to the slow turnaround time of conventional microbiological tests for detecting bacteremia in septic patients. Noscendo GmbH (Duisburg, Germany) has developed the CE/IVD pipeline DISQVER for clinical metagenomics testing based on cell-free DNA (cfDNA) from blood samples to address this issue.

**Methods:**

We conducted a retrospective study to evaluate the diagnostic utility of this methodological setup in improving treatment decisions since it was introduced into our clinical setting. Between January 2021 and June 2022, the first 300 cases in which DISQVER was applied at our university hospital were collected and analyzed. The results were compared with routine microbiology test results, clinical picture, associated treatment decisions, and clinical course.

**Results:**

Our findings demonstrate that DISQVER results where no pathogen was reported effectively ruled out bacterial bloodstream infections, whereas positive results varied in their usefulness. While the metagenomic approach proved highly valuable for detecting non-culturable and rare pathogens, its utility was limited in cases where detected microorganisms were commonly associated with the microbiota.

**Discussion:**

Performing on-site analysis might mitigate delays resulting from logistical challenges and might help optimizing antibiotic stewardship. Once prompt results can be obtained, the relevance of incorporating molecular resistograms will become more pronounced. Further, the specific patient subgroups that most benefit from this analysis must be worked out. Guiding clinicians in identifying the infection focus based on the detected bacteria would significantly improve patient care. Lastly, evidence of filamentous fungi must be diligently followed up.

## 1 Introduction

Despite the extensive range of antibiotics available in the 21st century, bacterial bloodstream infections remain among the most significant global challenges for intensive care units and diagnostic laboratories and cause substantial morbidity and mortality (Retamar et al., 2012; Lillie et al., 2013; McNamara et al., 2018; Timsit et al., 2020). In addition to the growing number of pathogens resistant to first-line antibiotics, a significant challenge is the lack of a timely diagnostic workup with sufficient sensitivity to identify causative microorganisms and their susceptibility (Retamar et al., 2012; Gutiérrez-Gutiérrez et al., 2017; Timsit et al., 2020). Both aspects are vital to significantly improve clinical outcomes of bloodstream infections, as timely administration of appropriate antimicrobial therapy is paramount for treating sepsis (Gutiérrez-Gutiérrez et al., 2017; Timsit et al., 2020; Asner et al., 2021). Blood cultures remain the most recognized microbiological tests for detecting bacteremia in septic patients; however, these can take several days to provide results (Loonen et al., 2014). Moreover, they are prone to contamination or false-negative results, mainly when collected after antibiotic therapy (Hall and Lyman, 2006; de Prost et al., 2013; Loonen et al., 2014). As a result, septic patients are typically treated with an empirical broad-spectrum antibiotic (combination), leading to a significant risk of antimicrobial overtreatment, antibiotic-induced toxicity, and selection of multidrug-resistant pathogens (Takamatsu et al., 2020; Bruns and Dohna-Schwake, 2022). Biological markers that indicate host’s endogenous response to infection are already widely used (Xie, 2012; Cho and Choi, 2014). Yet, this approach can only tell something about the presence of an infection, not about the infectious agent. Regarding the latter, various novel techniques were developed to improve or complement conventional methods and to recognize bloodstream infections earlier (Liesenfeld et al., 2014, B).

Next-generation sequencing of circulating cfDNA (cell-free DNA) from whole blood samples has recently become clinically available for sepsis diagnostics (Grumaz et al., 2016; Long et al., 2016; Grumaz et al., 2020). While this approach has the potential to provide valuable complementary input to conventional diagnostics, its impact is still to be determined. Starting in 2020, several German public health insurances have begun to cover the cfDNA-based pathogen detection method DISQVER developed by Noscendo GmbH (Duisburg, Germany). Intensive care physicians and microbiologists have since utilized this promising new diagnostic method across Germany. Nevertheless, evidence-based protocols outlining when and in which cases this analysis is most beneficial still need to be formulated. Intending to determine the clinical utility of this method, we retrospectively compared the DISQVER results with routine diagnostic results and treatment decisions in the first 300 cases in which DISQVER was applied at our University Hospital and assessed whether it enabled faster and/or more guided decisions.

## 2 Materials and methods

The UKB is a tertiary referral and maximum care hospital with over 1,200 beds. Our microbiological diagnostic unit services the entire hospital, but predominantly, the intensive care departments of the hospital used the DISQVER tests in the period under performance study assessment. For DISQVER samples blood was drawn into Streck blood collection tubes which were retrieved by courier upon request and processed off-site (Noscendo GmbH Laboratories, Reutlingen, Germany). Reports were sent at the earliest on the 2^nd^ day after sample collection, with shipping duration matching the time required for subsequent processing. Samples collected and shipped on weekends or national holidays were not processed before the subsequent working day.

Sample preparation and sequencing and analysis have been previously described (Blauwkamp et al., 2019; Brenner et al., 2021). The DISQVER platform comprises a curated microbial genome reference database of over 16 000 microbial species covering more than 1500 pathogens and can detect bacteria, DNA viruses, fungi, and parasites. The report comprises a list of microorganisms detected at clinically relevant levels for each analyzed sample, along with their respective read counts, and does not include a molecular resistogram, treatment recommendations, or other interpretative clinical guidance.

The first 300 DISQVER results generated in our hospital were retrieved from the online access platform and matched the results of blood cultures (BCs) collected on the same day (+/-24h). Blood cultures were incubated for up to 5 days in the Bactec FX blood culture system (Becton Dickinson, Heidelberg, Germany). For identification the Vitek MS (bioMérieux, Marcy l’Etoile, France) was used. DISQVER samples taken from the same patient and with the same results within 14 days were considered as duplicates, and only the first was considered for analysis.

In cases of DISQVER results positive for blood-culturable pathogens (BCP) the results of BCs collected on the same day were retrieved. In the case of matching BC results, it was recorded whether (and if so, by how much) DISQVER results were available faster than routine results and whether, based on that, treatment decisions were made/or could have been made more quickly. In cases without BC collected on the sampling date, results of BCs collected up to 72 hours earlier or later were retrieved, together with the results of other microbiological tests performed during that hospitalization, information on the clinical picture, and clinical course. Cases with DISQVER results positive for BCP but without matching BC or non-BC tests were screened on whether there was a modification in ongoing antibiotic therapy based on the DISQVER result and whether the DISQVER result could explain clinical improvement or lack thereof.

In DISQVER negative cases for BCP (negative or such only reporting viral pathogens), it was retrieved if BC were collected on the same day or at least up to 72 hours earlier or later and if and what growth occurred in these. DISQVER turnaround time was determined for all cases. Likewise, for all blood samples collected, both for blood cultures and cfDNA sequencing, the collection site and modality (e.g., central venous catheter, arterial catheter, or peripheral venipuncture) were documented.

All data relevant to the study are included in the article.

The University Hospital Bonn ethics committee confirmed that no ethics approval was required for this study.

## 3 Results

**Figure 1:**
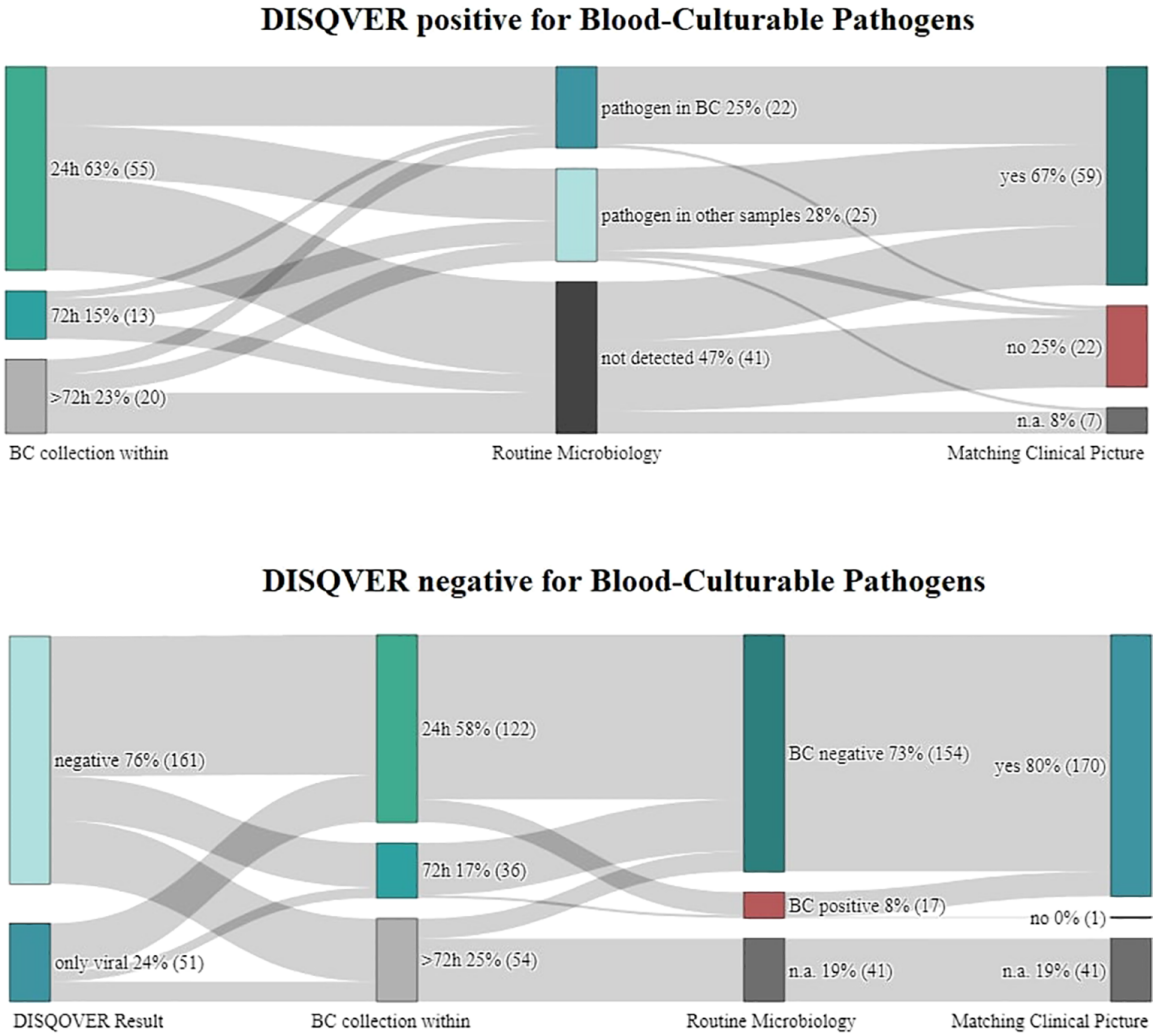
Display if and how often blood was drawn for cultures simultaneously as for microbial cell-free DNA (mcfDNA) sequencing and whether results of routine investigations and the patient’s clinical picture corresponded to DISQVER results.

Samples of the first 300 cases that met the defined selection criteria belonged to 248 patients and were collected between January 1st, 2021, and June 22nd, 2022. Another 51 samples were collected in that period but were considered as duplicates and were excluded. The median age was 58.6 years (range: 4-91 years), with 154 males (62.1%) and 94 females (37.9%). **Figure 1** shows how often DISQVER results matched routine microbiology and clinical picture.

In 161 cases, the DISQVER pathogen test was negative. In 51 cases, only viral DNA was detected. The remaining 88 cases were positive for blood-culturable pathogens (BCP). BCP-positive DISQVER results occur into four categories: 1) those confirmed by blood cultures (BC) collected on the same day (+/-24 hours); 2) those confirmed by BC collected earlier or later than DISQVER samples during that hospitalization; 3) those in which DISQVER pathogens were only found in other microbiological samples; and 4) those that could not be reconfirmed in routine diagnostics.

In 131 (43.66%) cases, the actual turnaround time matched the minimum expected duration of two days due to transportation and processing. These were exclusively cases in which samples were collected between Mondays and Thursdays. In 66 (22.00%) of cases reports were available after three days and only included such in which samples were collected between Sundays and Wednesdays. In the remaining 105 (35.00%) of cases, reports were sent within four days, primarily due to sample collection on Fridays, Thursdays, and Saturdays (in this order), or even later if national holidays further impeded transportation or processing. Turnaround time for Thursday samples depended on collection time: those collected in the late afternoon were shipped earliest on Friday resulting in mentioned delayed DISQVER reporting.

### 3.1 Negative DISQVER results

BCs were drawn within 24 hours of DISQVER sampling in 91 of the 161 DISQVER negative cases. In eight cases, there was growth of a microorganism in BC. Clinically seven were deemed contaminations at an early stage, and these grew *Corynebacterium amycolatum* (1/6 BC), *Cutibacterium acnes* (2/6 BC), *Enterococcus faecium* (1/6 BC), *Staphylococcus epidermidis* (1/6 BC), *Staphylococcus haemolyticus* (1/6 BC), *Staphylococcus hominis* (1/9 BC), and *Streptococcus anginosus* (1/6 BC). One, growing *Rothia mucilaginosa* (2/12 BC) presented an interpretative challenge but was ultimately classified as a colonization of the central venous catheter (CVC) rather than a bloodstream infection, leading to the replacement thereof.

In 29 cases, BC was not drawn within 24 hours but at least within 72 hours of DISQVER sampling. In one case, there was growth of *Staphylococcus aureus* in 1/23 BC. Subsequently, the patient was closely monitored, but no treatment was administered, and no adverse outcomes were observed.

### 3.2 DISQVER results only positive for viral pathogens

*Epstein-Barr virus*, *Human alphaherpesvirus 1*, *Human betaherpesvirus 6B*, *Human cytomegalovirus*, and torque teno viruses were the viral pathogens most commonly detected in this group of DISQVER results. BCs were drawn within 24 hours of DISQVER sampling in 31 of these 51 cases. In seven cases, microorganisms grew in BC. Clinically, all were deemed contaminations. In one case, out of 3 BCs collected from CVC, one was positive with *Candida albicans* and one with *Candida glabrata*. The patient received voriconazole in addition to meropenem and moxifloxacin due to a suspicion of nosocomial pneumonia and sepsis. Clinically, the findings of *Candida* were not considered relevant in the context of the pneumonia, and the anti-fungal therapy was not modified to better cover the *Candida* species, without adverse outcomes. The remaining BC featured growth of *Cutibacterium acnes* (1/6 BC), *Granulicatella adiacens* (1/3 BC), and thrice *Staphylococcus epidermidis* (1/6; 1/6; 2/6 BC).

In another seven cases, BC were not obtained within 24 hours but within 72 hours of DISQVER sampling. In one case, there was growth of *C. albicans* in 1/12 BC, yet the attending physicians clinically assessed it as either a contaminant or a result of potential sample mishandling.

Our investigation found that contamination was suspected in retrospect in 16 out of 17 cases where growth occurred in BCs that were DISQVER negative for BCP. However, due to the positive BC microscopy findings with gram-positive staphylococci, vancomycin treatment was initiated in two patients. In the 17th case (*C. albicans* in 1/12 BC), colonization of the CVC was deemed more likely than bloodstream infection. However, due to insufficient documentation, this could ultimately not be definitively confirmed.

### 3.3 DISQVER concordant with BC collected within 24h

**Figure 2:**
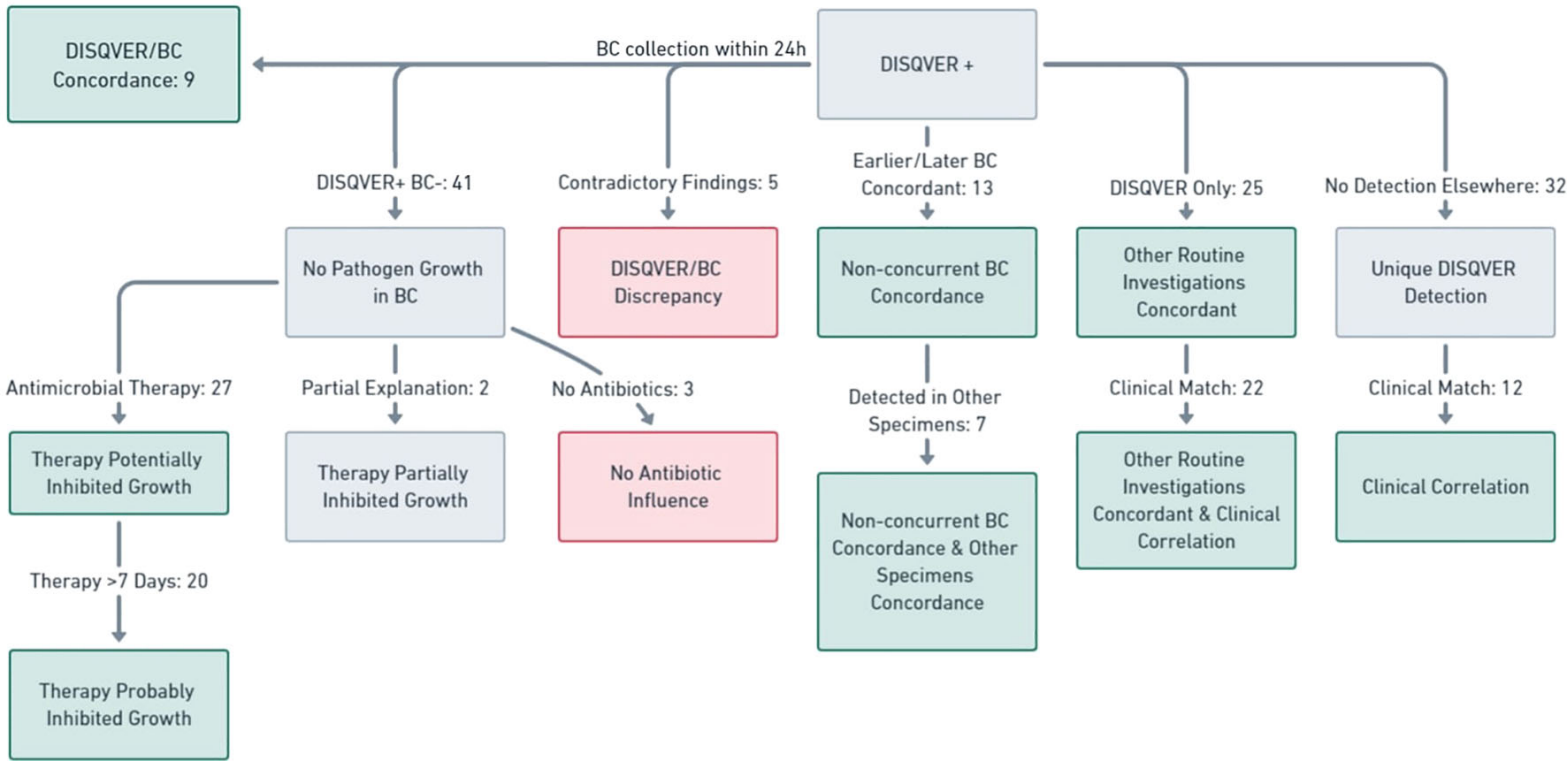
Comprehensive overview of the concordance between DISQVER positive results, BC, other routine investigations and the clinical picture.

In only nine cases out of the 55 (16.36%) in which BC were collected within 24 hours of DISQVER sampling, the pathogens detected by DISQVER grew in BC (see **Figure 2**). These were *Bacteroides fragilis* (1/2 BC), twice *Enterococcus faecium* (3/4; 4/6 BC), *Escherichia coli* (4/12 BC), *Pseudomonas aeruginosa* (1/6 BC), twice *S. aureus* (2/4; 5/8 BC) and *S. epidermidis* (12/12 BC). In one case, DISQVER reported *Candida parapsilosis* and *E. faecium*, where only *C. parapsilosis* grew in BC (3/8).

In four cases, the results of DISQVER testing were available within two days of sample collection; in one case, it took three days, and in another four cases (all four collected late on Fridays), it took four days. Gram-stain results were available sooner than the DISQVER results in six cases, with five of these cases also having identification via MALDI-TOF MS and phenotypical susceptibility profiles available earlier.

DISQVER only provided additional diagnostic information in one case, where it detected *B. fragilis*. As a result, the clinical microbiologist recommended adding metronidazole to the ongoing antibiotic therapy (meropenem), which seven days later turned out to be the only tested substance the isolate was susceptible to, as per the EUCAST 2024 v14 breakpoints (version 01.01, 2024).

### 3.4 DISQVER positive but BC collected within 24h negative

In 32/41 cases in which BC were collected within 24 hours of DISQVER sampling and the pathogens detected by DISQVER did not grow in BC, information on ongoing or prior antibiotic therapy was available. In 27 of these cases, the patients were receiving an antimicrobial therapy at the time the blood cultures were collected, that would likely have inhibited the growth of the respective pathogens detected by DISQVER (in 20 cases patients were on antimicrobial therapy for already >7 days). In two cases the ongoing and prior antimicrobial therapy only partially explained the lack of growth in BC. In three cases no antimicrobials were administered (see **Figure 2**).

### 3.5 Conflicting findings

In five cases, despite blood culture being collected on the same day, DISQVER and BC findings contradicted each other. In none of these cases, peripheral blood was collected for DISQVER. In four of these, *S. epidermidis* grew in BC (1/6; 2/6; 2/6; 4/11), whereas DISQVER reported 1) *Delftia* spp., *Pseudomonas* spp., *Burkholderia* spp., and *C. acnes*; 2) *Aspergillus fumigatus*, *Staphylococcus pasteuri*, and *S. hominis*, 3) *Lactobacillus* spp. and *C. albicans* and 4) *C. acnes*. All four *S. epidermidis* grew in BCs collected from CVC. In one case *Pichia kudriavzevii* (*Candida krusei*) grew in 3/7 BC but DISQVER reported *A. fumigatus*, *Aspergillus nidulans* and *C. parapsilosis*.

### 3.6 DISQVER concordant with earlier or later BC

In 13 additional cases, pathogens detected by DISQVER grew in BC that were collected markedly earlier or later than DISQVER sampling but during that same hospitalization. These are displayed in **Table 1**, and in three cases, 20 or more days separated DISQVER sampling and sampling of the concordant BC. In seven cases, pathogens were also detected in specimens other than BC. DISQVER often detected multiple pathogens simultaneously, but only some grew in blood cultures. In four cases, DISQVER pathogens with the highest number of reads were not those that grew in BC, but in two of these cases, those with the most reads grew in other clinical specimens. The clinical picture and course matched DISQVER results in all but one case.

**Table 1:** DISQVER results (with reads) confirmed by BC collected >72 hours earlier or later than DISQVER sampling and where applicable in non-BC routine microbiological samples.

DISQVER result with reads	The same species in other microbiological samples	24h*	72h*	CP
***Aspergillus fumigatus*** *: 68*	BC (+6)	-	3	✔
***Bacteroides fragilis*** *: 194*, *Klebsiella pneumoniae: 99, Phocaeicola vulgatus: 97*, ***Klebsiella oxytoca*** *: 66*, *Klebsiella aerogenes: 33*	2 BC (-12) - - 2 Ascites, TS (-11) -	-	6	✔
***Candida dubliniensis*** *: 652735*, *Candida albicans: 110*, *Ureaplasma parvum: 100*, *Candida tropicalis: 29*	NS (-20, -1), Stool (-15), Urine (-15, -5), BC (-12,-6), Wound (-8, -7, -4, -1), PlPu (-5) - - -	9	3	✔
***Enterococcus faecium*** *: 10*	2 BC (-42)	-	-	✔
***Enterococcus faecium*** *: 39*,	Bile (-15), 2 BC (-13), AbPu (-7, -6, +17), Wound (-6)	4	6	✔
***Enterococcus faecium*** *: 1201*, ***Candida albicans*** *: 4*	Wound (-10, -7, 0) 4 BC (-13)	-	-	✔
*Lichthehimia ramosa: 132* ***Enterococcus faecium*** *: 65*	- BC (+5)	-	-	✔
***Pseudomonas aeruginosa*** *: 418*	Stool (-19, +21), NS (-1, +7, +12), BC (+20, +38)	10	-	✔
*Rhizopus microsporus: 134* ***Staphylococcus haemolyticus*** *: 123*	- 3 BC (-6), CVC tip (-5)	3	3	✔
***Staphylococcus aureus*** *: 282*	3 BC (-8, -7, -6)	2	-	✔
***Staphylococcus epidermidis*** *: 6223*, *Enterococcus faecium: 1047*, *Staphylococcus haemolyticus: 437*, *Veillonella parvula: 151*, *Lactobacillus delbrueckii: 63*	2 BC (-6) - - - -	-	-	✔
***Staphylococcus epidermidis*** *: 204*, *Aspergillus fumigatus: 16*	25 BC (+62, +63, +64, +65, +66, +69) -	3	6	✔
***Enterococcus faecium*** *: 575*, *Staphylococcus haemolyticus: 108*, ***Staphylococcus epidermidis*** *: 29*, *Enterococcus faecalis: 10*,	Urine (-14, -3), Stool (-2) - BC (-3) -	3	3	x

CP, Clinical picture matches DISQVER result. Numbers in brackets refer to the days samples were collected before (-) or after (+) DISQVER sampling. (AbPu, abdominal punctation; BC, blood culture; CVC, central venous catheter; NS, nasopharyngeal swab; PlPu, pleural punctuation; TS, tracheal secretions). All microbiological samples collected during that respective hospital stay were considered.

*Number of blood cultures collected within 24 and 72 hours of DISQVER sampling.

✔ = matching clinical picture and x = not matching clinical picture.

In four cases, it was possible to discern from the case record that therapy was changed/extended based on the DISQVER evidence. The patients with DISQVER reports of *Lichthehimia ramosa* and *Rhizopus microsporus* were both in aplasia with radiological suspicion of a pulmonary focus. The former received a partial lung resection within a few weeks due to a mucormycosis. The latter’s liposomal amphotericin B dosage was increased. In both patients, clinical improvement occurred.

### 3.7 DISQVER pathogens only detected in other clinical samples

In 25 cases, DISQVER pathogen reports were not matched by BC results but by the results of other routine investigations. **Table 2** lists these cases. In 22/25 cases, clinical presentation matched the DISQVER results. In one case, additional data would have been required for appropriate evaluation but was not available. In 13 of these cases, it was possible to assess, based on existing records, whether the ongoing antibiotic therapy was modified exclusively due to the DISQVER report. This occurred in only one of these 13 cases but without clinical improvement.

**Table 2:** DISQVER results (with reads) only confirmed by non-BC diagnostic tests.

DISQVER result with reads	The same species in other microbiological samples	24h*	72h*	CP
***Aspergillus fumigatus*** *: 381*	BAL-PCR (-1), 3 TS (+3)	-	21	✔
***Aspergillus fumigatus*** *: 860* *Staphylococcus epidermidis: 50* *Enterococcus faecium: 8*	TS (-8, -6, -5, -1) - -	6	6	✔
***Candida albicans*** *: 103*	Wound (+15, +22, +32, +33)	-	-	✔
***Candida albicans*** *: 35*, ***Candida parapsilosis*** *: 4*	Wound (-38, -29, -8, -3), TS (+7) Wound (-5)	6	-	✔
***Candida dubliniensis*** *: 8402*, ***Candida albicans*** *: 11*	BAL (0) Stool (-7), Urine (-10, -11, -13)	9	-	✔
***Candida tropicalis*** *: 6*	AbPu (0)	-	6	✔
***Chlamydophila psittaci*** *: 812*	Serological Test (+29)	12	6	✔
***Citrobacter freundii*** *: 112*, ***Prevotella oris*** *: 12*, *Streptococcus milleri: 11*, *Streptococcus anginosus: 6*, *Streptococcus intermedius: 6*	TS (-1, 0) Abscess (-15) - - -	10	-	✔
***Citrobacter koseri*** *:11*	TS (-2, -1), BAL (0, + 2), Stool (+8), Urine (+25)	6	-	✔
***Cyclospora cayetanensis*** *: 41*	Anal –Swab-PCR (+3)	-	12	✔
***Enterococcus faecium*** *: 542*	Wound (-14, -11, -5)	-	-	✔
***Enterococcus faecium*** *: 19*	Joint Sonication (-19)	9	6	✔
***Enterococcus faecium*** *: 129*, *Bacteroides uniformis: 27*, *Bacteroides ovatus: 11*	Ascites (-2), AbPu (0) - -	9	-	✔
***Escherichia coli*** *: 13*	Bile (-7)	-	-	✔
***Legionella pneumophila*** *: 12*	Urine AG-Test (-1), TS (0), TS-PCR (0)	6	6	✔
***Klebsiella pneumoniae*** *: 121* *Enterococcus faecalis: 13*	BAL, BAL-PCR, TS (-2) -	-	12	✔
***Prevotella nigrescens*** *: 28* *Prevotella oris: 12* *Mycoplasma salivarium: 9*	Fascial Tissue (-4) - -	-	6	✔
***Pseudomonas aeruginosa*** *: 22654*	Wound (+1), Urine (+1, +2), Stool (+2)	6	8	✔
*Roseomonas gilardii: 14* ***Mucor circinelloides*** *: 3*	- TS (-7, -3, +3) BAL (-6, -2)	-	-	✔
***Serratia marcescens*** *: 34*, ***Enterococcus faecium*** *: 11*	TS (+2, +10, +23) Wound (-19, +20)	-	-	✔
***Staphylococcus aureus*** *: 148*	Sputum (-4), TS (+3)	-	6	✔
***Staphylococcus aureus*** *: 93*, ***Serratia marcescens*** *: 32*, *Neisseria flavescens: 22*, *Neisseria subflava: 12*	BAL (-1, 0), BAL-PCR (-1) BAL (-1, 0), BAL-PCR (-1) - -	6	-	✔
***Aspergillus fumigatus*** *: 90*, *Malassezia globosa: 7*	Aspergillus-Ag in TS (+29) -	6	5	x
***Enterococcus faecium*** *: 5*	Bile (+9)	6	-	x
***Enterococcus faecium*** *: 1477*, *Proteus mirabilis: 276*, ***Klebsiella pneumoniae*** *: 157*, ***Pseudomonas aeruginosa*** *: 34*, *Lactobacillus paracasei: 21*	Wound (0) Urine (-28) - Wound (0) Urine (-3, -1), Wound (0) -	6	-	-

CP, Clinical picture matches DISQVER result. Numbers in brackets refer to the days samples were collected before (-) or after (+) NGS sampling. (AbPu, abdominal punctation; BAL, bronco-alveolar-lavage; BC, blood culture; PlPu, pleural punctuation; TS, tracheal secretions).

*Number of blood cultures collected within 24 and 72 hours of DISQVER sampling.

### 3.8 DISQVER pathogens not found in routine diagnostics

In 32/88 positive cases, none of the pathogens identified by DISQVER were detected in any other microbiological tests. Therefore, it was crucial to correlate the results with the clinical presentation. For instance, imaging findings strongly suggested fungal pneumonia in all five cases in which DISQVER reported *Aspergillus* species. Two of these patients were switched to voriconazole and isavuconazole, respectively, resulting in significant improvement. Another case involved the detection of *Fusobacterium necrophorum* by DISQVER in a patient subsequently diagnosed with Lemierre’s syndrome. **Table 3** provides the DISQVER results, the consistency of the clinical presentation with the result, whether there was a modification in ongoing antibiotic therapy based on the DISQVER result, and whether the DISQVER result could explain clinical improvement or lack thereof.

**Table 3:** DISQVER results (with reads) not found in other diagnostic tests.

DISQVER result with reads	24h*	72h*	Matching clinical picture	Therapeutic shift	Explains outcome
*Acinetobacter junii: 15*	-	-	✔	✔	✔
*Aspergillus fumigatus: 130*	3	-	✔	x	✔
*Aspergillus fumigatus: 18*	-	9	✔	x	✔
*Aspergillus fumigatus: 21*	3	-	✔	x	x
*Aspergillus oryzae: 159*, *Aspergillus flavus: 64*, *Candida albicans: 30*	-	-	✔	-	-
*Bacteroides ovatus: 21, Bacteroides fragilis: 11*	12	-	✔	x	x
*Fusobacterium necrophorum: 1021*	2	-	✔	x	✔
*Klebsiella aerogenes: 6*	3	-	✔	x	✔
*Kocuria palustris: 78, Brevibacterium linens: 26, Lactobacillus reuteri: 21, Carnobacterium inhibens: 12, Brevibacterium aurantiacum: 8*	-	-	✔	✔	x
*Prevotella jejuni: 99* *Prevotella nigrescens: 27*	6	-	✔	-	-
*Pseudomonas aeruginosa: 49451, Escherichia coli: 749*, *Comamonas testosteroni: 455, Pseudomonas litoralis: 139, Klebsiella* sp.*: 124*,	3	-	✔	x	✔
*Streptococcus gordonii: 19, Capnocytophaga leadbetteri: 7*	-	6	✔	✔	x
*Acinetobacter haemolyticus: 14*	1	4	x	x	-
*Aureobasidium melanogenum: 3*	-	-	x	x	-
*Auricoccus indicus: 14*	6	-	x	x	-
*Bacteroides fragilis: 7*	6	-	x	x	-
*Burkholderia contaminans: 10*	6	-	x	x	-
*Cutibacterium acnes: 117*	6	3	x	x	-
*Cutibacterium acnes: 122*	3	6	x	x	-
*Cutibacterium acnes: 299, Burkholderia contaminans: 138*	2	-	x	x	-
*Enterococcus faecalis: 5*	6	6	x	x	-
*Enterococcus faecalis:bacteria:24, Klebsiella oxytoca:bacteria:13*	-	-	x	-	-
*Enterococcus faecium: 10*	6	-	x	x	-
*Enterococcus faecium: 20, Penicillium nalgiovense: 9*	9	-	x	x	-
*Enterococcus faecium: 7*	6	-	x	x	-
*Lactobacillus rhamnosus: 8*	15	7	x	x	-
*Penicillium nalgiovense: 18*	2	6	x	x	-
*Pseudomonas aeruginosa: 100, Staphylococcus epidermidis: 27, Klebsiella pneumoniae: 22, Staphylococcus pasteuri: 21, Enterobacter cloacae: 7*	-	-	x	-	-
*Staphylococcus epidermidis: 79*	-	3	x	✔	✔
*Staphylococcus hominis: 23*	-	-	x	-	-
*Phocaeicola dorei: 6*	-	6	-	-	-
*Serratia marcescens: 8*	-	6	-	-	-

✔, yes; x, no.

*Number of blood cultures collected on the day of NGS sampling or up to 72 hours earlier or later.

In Germany, hospital billing, usually via DRGs, for sepsis is dependent on the identification of pathogens, as the identification of a pathogen often results in more targeted and possibly more expensive treatment. In 57/88 of DISQVER-positive cases, i.e. 19% of all cases, DISQVER pathogen detection could have led to an increase in the diagnosis-related group (DRG) based per-case reimbursement.

## 4 Discussion

cfDNA circulating in plasma has mainly been used as a prognostic marker so far and has been appraised as a good predictor of patient outcome in ICU (Dwivedi et al., 2012; Ahmed et al., 2016; Volik et al., 2016; Chiu and Miller, 2019), less so as a marker of sepsis (Ahmed et al., 2016). However, rather than sequenced, cfDNA is only quantitatively assessed when used as a prognostic marker. Given that most circulating cfDNA in the blood is host-derived (Dwivedi et al., 2012), studies discarding the value of cfDNA in sepsis primarily refer to circulating cell-free host DNA. With metagenomic next-generation sequencing (mNGS) techniques, it is principally possible to sequence mentioned DNA in every routine molecular laboratory within hours (Chiu and Miller, 2019). When mNGS is applied to cfDNA, cfDNA is not only quantitatively assessed. Sequencing cfDNA generates high-quality data that can be used for purposes ranging from tumor detection (Volik et al., 2016) to pathogen identification (Forsblom et al., 2014; Grumaz et al., 2016; Long et al., 2016; Hong et al., 2018; Huang et al., 2018; Grumaz et al., 2020; Wang et al., 2021), as in our study.

Rather than human DNA, in sepsis diagnostics, most of the interest is limited to sequenced microbial DNA. However, due to technical considerations, human DNA is generally sequenced alongside and only filtered out bioinformatically. As a result, the term cfDNA sequencing is often used when only microbial cell-free DNA (mcfDNA) is evaluated. Attention is therefore warranted when comparing studies evaluating the clinical potential of measuring cfDNA, sequencing cfDNA, and sequencing cfDNA for pathogen detection. Plasma mcfDNA testing has the potential to identify, in a hypothesis-free manner, a broad spectrum of infections throughout the body and inform clinicians beyond the classic manifestations of infectious disease. The results of our study, however, highlight that in our setting, the method does not yet seem to live up to its potential.

Studies involving from tens to hundreds of subjects have attempted to evaluate the sensitivity (70.0%-92.9%) and specificity (62.7%-88.2%) of mcfDNA sequencing for pathogen identification, using the results of conventional methods and/or clinical judgment as reference standards (Han et al., 2020). We did not aim to determine the sensitivity or specificity of the test. However, it should be noted that in no DISQVER-negative sample (and those only reporting viruses), there was cultural growth of a microorganism that was undoubtedly considered the causative pathogen. To truly capture the sensitivity, it would be beneficial to compare the performance of DISQVER and cfDNA sequencing in general, in addition to culture and clinic, also to metagenomic tests that also target/include intracellular DNA. Given the hypothetical relatively short turnaround time, if the high sensitivity were confirmed, the test would prove very valuable in ruling out an infection.

In this study, it was not possible to reliably assess whether DISQVER-negative results lead to an antibiotic de-escalation, which should be addressed in future studies. In our study, clinicians mainly called upon DISQVER when all other diagnostic options had been exhausted, but the patient was still suspected of having an infectious condition. However, the strength of metagenomics may lie in its potential for early exclusion of infections, which could yield significant benefits in antibiotic stewardship.

Another strength lies in detecting pathogens that are difficult or slow to grow, unexpected, or already treated with antibiotics; this advantage is common to all metagenomic approaches and has been widely documented (Gu et al., 2019; Han et al., 2020; Freeman Weiss et al., 2021). Examples from our study include *Chlamydophila psittaci*, *Cyclospora cayetanensis*, *Fusobacterium necrophorum*, and various *Aspergillus* spp. results in combination with radiological suspicions of fungal pneumonia. In retrospect, the detection of filamentous fungi almost invariably aligned with the clinical presentation and progression, underscoring their identification as particularly significant. The inclusion of parasites and viruses within the scope of detection significantly broadens the diagnostic potential, offering capabilities that extend well beyond the confines of traditional blood culture methodologies. Notably, from an accounting perspective, the identification of such pathogens, including those that may have been suppressed by antibiotic treatment, entitled to increased DRG-based per-case reimbursement in 19% of cases.

In addition to its use for sepsis diagnosis, sequencing cfDNA has also been discussed for preventive pathogen detection before the onset of bloodstream infections in particularly vulnerable patient groups (Goggin et al., 2021). Furthermore, cfDNA sequencing also holds interesting potential when applied to diagnose invasive infections beyond sepsis and using materials other than blood (Yu et al., 2022; Li et al., 2023; Hu et al., 2024; Petri et al., 2024). Our study encountered instances where DISQVER identified pathogens that were only later detected in blood cultures. However, more commonly, we observed reports that presented challenges in interpretation for attending physicians, particularly in cases where multiple intestinal or skin pathogens were detected. In this regard, an intriguing breakthrough could be the suggestion of a probable infection focus based on evidence of specific pathogen patterns. Additionally, it warrants investigation whether the identification of torque teno viruses or other viruses could serve as a marker for excessive patient immunosuppression (Redondo et al., 2022). The most conflicting cases, all from DISQVER samples collected from CVCs, suggest a strong recommendation to avoid sampling from CVC.

Our study’s findings indicate that the processing time is a significant obstacle when using the DISQVER method in practice. This is not due to the method itself but is caused by logistical and operational issues. In most cases where the DISQVER results were concordant with those of the blood culture, the DISQVER report was available later than the microscopy results and, in some cases, even later than the preliminary and final results of antimicrobial susceptibility testing. This was particularly true when samples were obtained during weekends. Thus, the test would seem more suitable for sepsis diagnosis when it can be performed on-site, when needed, or at least daily, as the entire sample preparation and sequencing process for the DISQVER method can be conducted in most advanced laboratories equipped for molecular diagnostic testing, utilizing sequencing platforms that are increasingly available in microbiology departments and are often already established in molecular pathology and human genetics laboratories. Also, it is crucial to recognize that knowledge of the pathogen causing an infection does not directly dictate treatment choice. Hence, the ability to identify resistance genes and, ideally, to generate molecular antibiograms beyond resistance genes will be critical. However, this aspect will only become relevant once rapid results can be obtained.

Limitations of the study include its retrospective single-center design and the absence of an in-depth cost-benefit analysis. Additionally, comparing DISQVER to other NGS-based and non-NGS-based methods would enhance the assessment of its clinical utility.

## 5 Conclusion

Logistical delays currently compromise one of the main advantages that molecular genetic tests have over slow blood culture diagnostics. Performing on-site analysis could remedy this and maximize the potential of metagenomics to rapidly rule out infections and support antibiotic management. It is important to determine exactly which patient groups would benefit most from this relatively costly method in order to integrate it seamlessly into regular diagnostic procedures. Although its utility may be limited if the identified microorganisms are largely part of the body’s normal microbial community, the occasional detection of relevant pathogens missed by conventional methods has led to benefits in diagnosis, treatment and cost-effectiveness.

## Data Availability

The original contributions presented in the study are included in the article/supplementary material. Further inquiries can be directed to the corresponding author.
